# Non-immune Traits Triggered by Blood Intake Impact Vectorial Competence

**DOI:** 10.3389/fphys.2021.638033

**Published:** 2021-03-02

**Authors:** Octavio A. C. Talyuli, Vanessa Bottino-Rojas, Carla R. Polycarpo, Pedro L. Oliveira, Gabriela O. Paiva-Silva

**Affiliations:** ^1^Instituto de Bioquímica Médica Leopoldo de Meis, Universidade Federal do Rio de Janeiro, Rio de Janeiro, Brazil; ^2^Instituto Nacional de Ciência e Tecnologia em Entomologia Molecular, Rio de Janeiro, Brazil

**Keywords:** tolerance, vector competence, blood-feeding, immunity, pathogens, parasite–vector interaction, insect physiology, midgut homeostasis

## Abstract

Blood-feeding arthropods are considered an enormous public health threat. They are vectors of a plethora of infectious agents that cause potentially fatal diseases like Malaria, Dengue fever, Leishmaniasis, and Lyme disease. These vectors shine due to their own physiological idiosyncrasies, but one biological aspect brings them all together: the requirement of blood intake for development and reproduction. It is through blood-feeding that they acquire pathogens and during blood digestion that they summon a collection of multisystemic events critical for vector competence. The literature is focused on how classical immune pathways (Toll, IMD, and JAK/Stat) are elicited throughout the course of vector infection. Still, they are not the sole determinants of host permissiveness. The dramatic changes that are the hallmark of the insect physiology after a blood meal intake are the landscape where a successful infection takes place. Dominant processes that occur in response to a blood meal are not canonical immunological traits yet are critical in establishing vector competence. These include hormonal circuitries and reproductive physiology, midgut permeability barriers, midgut homeostasis, energy metabolism, and proteolytic activity. On the other hand, the parasites themselves have a role in the outcome of these blood triggered physiological events, consistently using them in their favor. Here, to enlighten the knowledge on vector–pathogen interaction beyond the immune pathways, we will explore different aspects of the vector physiology, discussing how they give support to these long-dated host–parasite relationships.

## Introduction

For a long time, the traditional thinking on the insect vector–pathogen interaction described this relationship as impinging a low or even no fitness cost to the insect host (Sisterson, 2009; Powell, 2019). However, the discovery of the insect immune system brought to the scene a plethora of immune genes that were frequently modulated by the infection and, in several cases, the upregulation of these genes promptly reduced pathogen burden (Saraiva et al., 2016; Shaw et al., 2018; Telleria et al., 2018; Matetovici et al., 2019; Salcedo-Porras and Lowenberger, 2019). These findings led to a large number of studies on the immune signaling pathways and immune effector genes capable of decreasing or blocking pathogen burden and vector competence, which could lead to the possibility of limiting the disease prevalence under field conditions.

The immunology conceptual framework that has dominated the last century is the idea that the capacity to eliminate the infectious agent is the hallmark of the organism’s reaction against the infection. This so-called pathogen resistance and the path that eventually leads to health are considered synonymous with the elimination of the infectious agent. However, in the last decade, several studies have directed attention to the ability of organisms to reduce deleterious effects of infections without relying on the elimination of the parasite, but rather acting by alternative mechanisms that could restore tissue homeostasis, reducing self-inflicted damage caused by the host immune reaction or by the pathogen directly (Chovatiya and Medzhitov, 2014). Some early studies have termed this disease endurance (Casadevall and Pirofski, 1999; Graham et al., 2005; Ayres et al., 2008). Later these processes directed to reduce fitness loss due to infection were grouped under the concept of disease tolerance (Medzhitov et al., 2012). The tolerance to disease is highlighted here as a way to fight infection. Mechanistic studies show that a wide variety of processes are implicated in disease tolerance, such as stress response, tissue homeostasis, wound repair, and energy metabolism (Shaw et al., 2018). Essentially, this fresh outlook in immunology changes the microbe-killing focus of the immune response to the promotion of host health and survival through a global regulation of physiological responses (Lissner and Schneider, 2018). In insects, these studies were mainly performed with model insects, especially *Drosophila* (Lissner and Schneider, 2018) and the concept of disease tolerance has not yet been explored in the context of the interaction between insect vectors and the pathogens they transmit, as recently pointed (Oliveira et al., 2020).

Blood-feeding is a central event in the life cycle of both the insect vector and the pathogens they transmit. Typically, few parasites or viruses are taken up by the insect along with a blood meal. The number of pathogens in the blood meal is known as the critical bottleneck that will define the success of the infection. After a meal, the midgut becomes an aggressive environment, quickly populated by digestive enzymes that can potentially attack the pathogen. Moreover, it is colonized by an exuberant indigenous microbiota that may be a competitor for the incoming invader, at a clear numerical disadvantage. In addition, like all epithelial tissues, one of the main functions of the gut epithelium is to be a barrier of protection from the external world and to select what should enter into the organism. For most hematophagous insects, blood is the essential source of amino acids used to make yolk proteins. Therefore, hormonal control of reproduction is usually triggered by blood intake, which is tightly linked to the pace of blood digestion (Hagedorn, 2004). Several reports have shown hormonal effects on parasite infectivity, which, in some cases, have been attributed to a crosstalk between hormone signaling cascades and immune pathways (Nunes et al., 2020). Finally, for most blood-sucking insects, vertebrate hosts might be available more than once in a lifetime, and the effect of multiple blood meals have been shown to affect the life of pathogens by mechanisms that are not fully understood (Serafim et al., 2018). To summarize, the physiology of blood digestion of the hematophagous insect is the actual landscape where pathogens transmitted by them will thrive or not, a concept that was also revised by Nouzova et al. (2019). More importantly, the functioning of these processes ultimately defines the fitness cost of the infection to the insect vector, a major variable that affects vectorial capacity. Here we have addressed just some topics of vector physiology to illustrate the general concept that the performance of host organism integrated basic functions is critical to the success of the vector/pathogen association. As these are multiple and comprehensive topics, our intention is not to exhaust the analysis of the literature related to them. More than that, our goal is to highlight the need of examining the role of these and other non-immune basic aspects of insect physiology, not reviewed here, in determining the vector competence.

## Blood Digestion and Metabolic Signaling

Some hematophagous arthropods feed on blood for their whole life cycle such as ticks and triatomine bugs, while in others, like mosquitoes and sandflies, only female adults feed on blood. In the adult stages, blood meals are strictly essential for oogenesis for most species. However, even for those few autogenous insects that use their teneral reserves to make the first batch of eggs, the following cycles of ovarian growth rely on vertebrate blood, and therefore, reproductive success depends on blood-feeding. The ingestion of a blood meal elicits a response in the gut-brain axis known to involve the central nervous system, the enteric nervous system, and the gastrointestinal tract (Hagedorn, 2004). The gut-brain crosstalk not only ensures the proper maintenance of gastrointestinal homeostasis but has multiple effects on insect physiology through neural, endocrine, immune, and humoral links (Gonzalez et al., 1999; Brown et al., 2008; Castillo et al., 2011; Gulia-Nuss et al., 2011). The nutritional intake connects intermediary metabolism to sexual maturation, oogenesis, microbiota colonization, and immune response, the latter being triggered by the encounter of the vector with pathogens.

Strictly speaking, digestion starts with hydrolysis of food by a vast array of digestive enzymes secreted after a meal (proteinases, carbohydrases, and lipases) that, together with nutrient transporting proteins, are needed to process nutrients in the gut (Santiago et al., 2017). However, in the context of hematophagous insects, proteases have received far more attention because blood is composed mainly of proteins (90% of dry weight), leading those insects to translate an arsenal of proteases to support protein digestion (Lehane, 2005; Brackney et al., 2010; Henriques et al., 2017; Sterkel et al., 2017). In most insects, proteolysis is based on trypsin and other serine proteases, in contrast to triatomines and ticks, where aspartic and cysteine proteases drive protein digestion (Sojka et al., 2008; Santiago et al., 2017; Henriques et al., 2021). Initially, protein degradation was described as accomplished by a few enzymes of each type, but genome sequencing in mosquitoes, triatomines, sandflies and ticks showed the existence of multiple copies of enzymes in all classes, revealing extensive gene duplications that probably occurred after the acquisition of the blood-feeding habit, suggesting the need for redundancy or some type of functional differentiation among enzymes of the same family (Sojka et al., 2008; Henriques et al., 2017; Santiago et al., 2017).

The possibility of pathogens being targeted by digestive enzymes and/or the manipulation of the host’s digestion process by the pathogens has been addressed in several studies, but most of them were performed before the genomic expansion of digestive proteases was acknowledged. Insect digestive tracts vary extensively in morphology and physicochemical properties, factors that greatly influence the potential interaction between their also diverse set of transmissible pathogens and digestive milieu. The activity of proteolytic enzymes in the gut of *Anopheles* mosquitoes does not appear to be affected by *Plasmodium* infection. In addition, differences in the activity of digestive proteases in general are not observed among mosquitoes with different levels of susceptibility to infection (Feldman et al., 1990; Chege et al., 1996; Jahan et al., 1999). However, proteolytic activities present in the midgut lumen are required to activate a chitinase secreted by these parasites that is essential in the initial event of midgut infection (Shahabuddin et al., 1993). In the *Leishmania mexicana-Lutzomyia*’s pair, the pathogen promotes a decrease of trypsin activity in the vector’s gut, increasing parasite burden. Trypsin knockdown exerts the same effect (Sant’Anna et al., 2009), probably making the parasite more resistant to the highly degradative habitat of midgut lumen. In contrast, in the triatomine bug *Rhodnius prolixus*, the activity of cathepsin D-like enzymes increases upon infection by *Trypanosoma cruzi* (Borges et al., 2006). Still, its inhibition did not affect parasite development in the conditions tested (Garcia and Gilliam, 1980). As we can see, the existing examples concerning proteases in the vector-parasite interaction were based on the analysis of total enzymatic activities, which resulted from the action of multiple enzymes belonging to the same class. These facts might be the answer to the diversity of responses observed among the different groups of insects. It would be interesting to know what are the specific proteases involved in the different events and if they can have the same role in the different vector-parasite pairs.

For enveloped viruses, it is known that the establishment of a successful infection is highly dependent on the fusion of the viral envelope with host cell membranes, where the envelope proteins need to be activated by proteolytic processing by host cell proteases (Klenk and Garten, 1994). As for *Aedes aegypti* infection by dengue virus, treating the mosquito with a trypsin inhibitor before exposure to the virus decreases the midgut infection, which can be partially rescued when the virus is previously incubated with bovine trypsin (Molina-Cruz et al., 2005). In this case, the authors discuss the possibility of the virus be pre-processed by trypsin before gut epithelia invasion, enhancing its virulence. In contrast, another work published in 2008 showed that silencing the late trypsin 5G1 or the addition of soybean trypsin inhibitor to the infectious blood meals increased midgut infection rates by DENV-2 (Brackney et al., 2008). The latter work was confirmed by a study showing that prior colonization of *Ae. aegypti* with the fungus *Talaromyces* sp. induced the downregulation of many digestive enzymes, including several trypsins, resulting in higher susceptibility to dengue infection. Moreover, knockdown of these trypsin genes was able to recapitulate the fungus-induced decrease in viral infection (Angleró-Rodríguez et al., 2017b). Although controversial, the studies made with dengue and *Ae. aegypti* suggest that blood digestion mediated by trypsins may influence the rate of DENV-2 infection. Although the literature on the subject is scarce, proteases may influence vector infection by viruses. The determination of the precise time course of viral invasion and protease expression together with the repertoire of proteases in the mosquito midgut would allow for a more comprehensive approach of the contribution of each digestive enzyme for mosquito vectorial competence.

There is now a general agreement that the successful infection of an insect vector involves a tripartite interaction between the insect, the pathogens and the intestinal microbiota (Cirimotich et al., 2011; Ramirez et al., 2012), a concept that has been verified for different host/pathogens associations (Castro et al., 2012; Narasimhan and Fikrig, 2015; Romoli and Gendrin, 2018; Telleria et al., 2018). Interestingly, studies performed with mammalian models revealed that proteases both from the host or from the microbiota are important modulators of intestinal homeostasis and are involved in the interaction with pathogens (Buzza et al., 2010; Motta et al., 2019; Edogawa et al., 2020; Kriaa et al., 2020). Therefore, it is tempting to speculate if the influence of proteases on vector infection by pathogens is not at least partially mediated by a role of these enzymes on intestinal microbiota.

In addition to proteins, vertebrate blood is also enriched with lipids. Host lipid usage by pathogens and regulatory changes of lipid metabolism triggered by infection appear as key players in several host-pathogen relationships, being essential determinants for vector competence, as recently revised by O’neal et al. (2020). Digestive lipases, such as TG lipases, are flux generating enzymes for lipid metabolism pathways. The essential process of absorption of digested lipids from the blood meal by midgut cells is followed by an increased lipid transport from the midgut to other tissues, to support development and oogenesis. This lipid transfer is promoted by lipophorin, the main hemolymph lipoprotein, and results in lipid accumulation in the fat body (revised by Gondim et al., 2018). Interestingly, *Plasmodium* oocysts in the *Aedes* gut basal lamina hijack mosquito lipophorin to support the parasite development (Atella et al., 2009) and knockdown of lipophorin by RNA interference (RNAi) strongly restricted development of *Plasmodium* oocysts, reducing their number by 90% (Cheon et al., 2006; Rono et al., 2010). Similarly, induction of lipophorin synthesis is observed in *C. quinquefasciatus* with the filaria *Wuchereria bancrofti* (Kumar and Paily, 2011).

Host lipid remodeling is also observed in vertebrate infection by arboviruses to support their replication (Ng et al., 2008; Fernández de Castro et al., 2016; Leier et al., 2020). Similar reprogramming events have already been shown in vector cells (Perera et al., 2012; O’neal et al., 2020). An increase in the number of lipid droplets has been observed in *Aedes albopictus* C6/36 cells infected with dengue virus (Samsa et al., 2009). Furthermore, lipidomic analyses of the same DENV-infected cells revealed a large number of differentially expressed genes of diverse classes of lipids such as phospholipids and sphingolipids (Perera et al., 2012). On the same way, *Ae. aegypti* Aag2 cell line showed a regulation in the expression of lipid-related genes upon dengue 2 infection (Barletta et al., 2016). Besides, *in vivo* lipidomics showed that dengue infection in *Ae. aegypti* mosquitoes changed the lipid profile, mainly based on the inhibition of acylglycerolphosphate acyltransferase (AGAPT1), leading to an accumulation of phospholipids that support the viral replication (Chotiwan et al., 2018; Vial et al., 2019).

The blood-fed *Ae. aegypti* gut seems to increase the expression of many lipid-related genes, such as fatty acid synthase and perilipin-like proteins, which boost lipid droplet formation after blood meal (Barletta et al., 2016). Lipid droplets were shown in mammals to serve as a signaling platform involved with the synthesis of bioactive lipids (eicosanoids) (Vallochi et al., 2018). In *Aedes* mosquitoes, it has been shown that the midgut epithelia synthesize prostaglandins in response to the microbiota expansion in a phospholipase A-dependent manner that tunes the innate immune system against viral infection (Barletta et al., 2020). Similarly, *Anopheles gambiae* midgut produces prostaglandins in response to microbiota elicitors upon *Plasmodium* invasion, which triggers a cellular immune response (Barletta et al., 2019). The bug *R. prolixus* humoral and cellular responses seem to be modulated by eicosanoids as well (Azambuja et al., 2017). Using *T. rangeli* infection model, it was demonstrated that the insect reduces the arachidonic acid (the eicosanoid precursor) circulating in the hemolymph, leading to an inhibition of hemocytes phagocytic activity in the hemolymph (Garcia et al., 2004b; Figueiredo et al., 2008). Although eicosanoids/prostaglandins are part of the immune molecular arsenal in insects, these arthropods lack the canonical cyclooxygenase (COX), a key enzyme to convert the arachidonic acid into eicosanoids (Varvas et al., 2009). This is intriguing because those insects respond to the treatment with pharmacological COX inhibitors, such as indomethacin and acetylsalicylic acid, impairing the eicosanoid synthesis and modulating the immune response (Garcia et al., 2004a; Barletta et al., 2020). A question that remains open is what are the enzymes that play the role of canonical vertebrate COX in insects. In this sense, a specific peroxinectin named Pxt, that catalyzes the formation of the prostaglandin H2 (PGH_2_), was identified in the follicle cells of *Drosophila* (Tootle et al., 2011). Later the same COX-Like activity was identified in the moth *Spodoptera exigua* (Park et al., 2014). Additionally, Barletta et al. (2019) showed that *An. gambiae* heme peroxidases 7 and 8 are important enzymes to synthesize the prostaglandin by the mosquito gut epithelia, suggesting possible candidates for alternative enzymes with COX-like activities.

The interplay between the lipid metabolism pathways and the autophagy-related molecular machinery, named as lipophagy, and its role in physiological and pathological processes in mammals has received increased attention in the last few years (Schulze et al., 2017; Kounakis et al., 2019). Recently, it was demonstrated that the Chagas’ disease vector *R. prolixus* can use lipophagy during starvation to increase life span and locomotor activity (Santos-Araujo et al., 2020). It would be interesting to verify how this lipophagic machinery works under infection by either *T. cruzi*, a parasite limited to the intestinal environment, or *T. rangeli*, which is capable to invade the hemocoel and colonize salivary glands. Moreover, in enteric-infected *Drosophila*, an immune response is assembled by a lipophagy-dependent activation of DUOX (Lee et al., 2018). Thus, the contribution of lipophagy to the success of vectors’ infection by their respective pathogens, remains obscure and deserve to be investigated.

Insects do not synthesize cholesterol *de novo*, meaning that this lipid has to be absorbed from the diet over their lifetime (Clark and Block, 1959; Zande, 1967). It has been shown in mammalian models that different immune challenges entail cholesterol mobilization (Tall and Yvan-Charvet, 2015). In the case of viruses, the infection interferes in several aspects of cholesterol metabolism, needed for the formation of cell membranes and intimately related to both the entry of viral particles in the cell and their exportation (Osuna-Ramos et al., 2018). Unfortunately, literature on vector biology only tangentially looked at this particular aspect, pointing out some genes involved in cholesterol metabolism and cellular traffic, such as the Niemann Pick 1 protein and the Sterol Carrier Protein 2, as host factors that allow the viral multiplication in the mosquito (Junjhon et al., 2014; Jupatanakul et al., 2014, 2017; Fu et al., 2015; Chotiwan et al., 2018). Dengue infection blocking by *Wolbachia* in mosquitoes also correlates with changes in cholesterol metabolism, trafficking, and accumulation (Geoghegan et al., 2017). Moreover, even though cholesterol is known as a precursor for the hormone ecdysone (Canavoso et al., 2001), the association between viral infection and hormonal signaling is largely unknown. Nevertheless, definitive evidence showing the contribution of dietary cholesterol to the viral replication in mosquito is still lacking. Additionally, it is also unexplored how serum cholesterol fluctuation in populations from endemic areas could correlate to the viral transmission by mosquitoes.

Some medical relevant parasites such as Apicomplexan and Trypanosomatids, similarly to insects, lack the capacity of *de novo* cholesterol synthesis (Coppens, 2013; Pereira et al., 2015). In order to differentiate and proliferate in the insect gut, they obtain cholesterol from the vertebrate’s plasma low-density lipoprotein (LDL) (Labaied et al., 2011; De Cicco et al., 2012; Petersen et al., 2017). Lipophorin, mentioned previously to be hijacked from mosquitoes by parasites, might be the lipoprotein responsible for cholesterol import during the parasite insect stage. However, this hypothesis remains to be tested.

Most articles that compare carbohydrate metabolism of vectors-fed in sugar-rich diets with those fed on blood have focused on the fat body and physiological homeostasis. Sugar metabolism in these animals is controlled at the hormonal level by ILP and juvenile hormone (JH) (Clifton and Noriega, 2011; Hansen et al., 2014; Hou et al., 2015; Roy et al., 2015). It is increasingly clear that parasites can dramatically change the cellular energy metabolism of their arthropod vectors, as recently revised by Samaddar et al. (2020). Still, for mosquito-arbovirus interactions, the knowledge on such metabolic alterations is limited. An *in vitro* study showed that Zika virus infection in *Ae. albopictus* drives the glucose metabolism toward the pentose phosphate pathway, differently from human cells that increase flux to the tricarboxylic acid cycle (Thaker et al., 2019). Activation of the pentose pathway provides NADPH for antioxidant pathways, which control the intracellular redox state. Maintaining the redox balance would be beneficial for viruses as it would protect them from oxidative damage. However, the relevance of these changes to the course of viral infection has not been experimentally addressed in the literature yet, despite alterations in expression of metabolic enzymes being regularly observed in transcriptomic analyses of infected vector digestive apparatus (Padrón et al., 2014; Angleró-Rodríguez et al., 2017a; Etebari et al., 2017; Narasimhan et al., 2017; Coutinho-Abreu et al., 2020). A large amount of gene expression data on vector infection has now accumulated, and it could be used to direct studies focusing on the crosstalk between canonical immunity pathways and carbohydrate/energy metabolism, the so-called immunometabolism, and the relevance of them to vector biology (Samaddar et al., 2020).

As in other organisms, in addition to its digestive role, the digestive tract functions as a nutrient sensor. Intestinal signaling is involved in integrative processes that link nutritional availability with behavior and metabolism and the microbial intestinal world, including eventual pathogens that come along with the food. One of the few reports on this subject showed that in *Ae. aegypti* blood intake triggers nutrient-sensing signaling, such as the Target of Rapamycin (TOR), responsible for translation of early trypsin (Brandon et al., 2008). This is extremely important for the course of digestion, as this initial event coordinates the late digestive phase (Barillas-Mury et al., 1995; Brackney et al., 2010). A broad spectrum of cellular mechanisms depends on a signaling pathway. The routes taken will rest on the pairs of signaling molecules and receptors that trigger the process. In addition to nutrients such as amino acids and heme, which act also as signaling molecules (Hansen et al., 2004; Oliveira et al., 2011a; Bottino-Rojas et al., 2015; Short et al., 2017), a blood meal brings also regulatory peptides that act as neurochemicals and hormones, like vertebrate insulin, insulin like growth factor (IGF1), TGF-β and other cytokines. Furthermore, the presence of parasites in the blood meal can antagonize or potentiate the effects of these vertebrate-borne signaling molecules in the vector organism (Pakpour et al., 2013a). The resulting cellular responses can be beneficial or detrimental to pathogen development. Most of the studies on signaling pathways and vector susceptibility/resistance to infection have focused on their role in the activation of immune pathways. These studies have been extensively discussed previously by others (Pakpour et al., 2013a, 2014; Urbanski and Rosinski, 2018; Sharma et al., 2019; Nunes et al., 2020). Among the best-known signaling pathways in insect vector species is the insulin/insulin-like growth factor signaling pathway (IIS), which is involved in the regulation of growth, longevity, reproduction and immunity. *An. stephensi* stimulated with human ILPs induces ROS-mediated signaling, without oxidative damage that culminates with NFκB inhibition, allowing the *Plasmodium falciparum* oocyst development (Surachetpong et al., 2011; Pakpour et al., 2012). On the other hand, dietary insulin showed a negative impact on flavivirus replication in *Ae. aegypti* and *Ae. albopictus* cells, and *Culex quinquefasciatus* adult mosquitoes, in a mechanism dependent on JAK/Stat activation (Ahlers et al., 2019). Furthermore, it was shown that insulin receptor knockdown in *C. quinquefasciatus* blocks filarial parasite development (Nuss et al., 2018).

The IIS pathway comprehends two branches: the mitogen-activated protein kinase (MAPK) and the phosphatidylinositol 3-kinase (PI3K)/Akt. A series of studies have shown that both branches are modulated by host blood components. Host growth factors/cytokines affect the mosquito-malaria parasite interaction by modulating the MAPK signaling pathway. Ingested human IGF1 reduces phosphorylation of the MAPK ERK signaling protein in *An. stephensi* midgut and decrease the intensity and prevalence of *P. falciparum* infection (Drexler et al., 2013). Accordingly, the mammalian host TGF-β-1 induces the expression of nitric oxide synthase and reduces the prevalence of *Plasmodium* infection in *An. stephensi*. This effect is inhibited by the activation of ERK (Surachetpong et al., 2009). Interestingly, TGF-β also appears to be critical for the survival of parasites such as *T. cruzi* and *Leishmania amazonensis* in mammalian hosts (Barral-Netto et al., 1992; Ming et al., 1995; Omer et al., 2000). However, the impact of host TGF-β on the interaction of these parasites with their vectors has not yet been investigated.

Regarding the PI3K/AKT, Corby-Harris et al. (2010) showed that the overexpression of an activated form of Akt in *An. stephensi*, a regulator of IIS, shortened the mosquito lifespan and increased resistance to *P. falciparum*. Lately, the same group showed that the sustained Akt activation in the mosquito midgut resulted in mitochondrial dysfunction coupled to Akt-mediated repression of autophagy and compromised midgut epithelial structure. The perturbation of midgut homeostasis enhanced parasite resistance and decreased mosquito lifespan (Luckhart et al., 2013).

Insulin-like peptides induced by blood-feeding trigger vitellogenesis in the fat body of *Ae. aegypti* and act as regulators of the blood digestion in the gut (Gulia-Nuss et al., 2011; Roy and Raikhel, 2012). In *An. stephensi*, ILPs interfere in the mosquitoes intermediary metabolism and nutrient intake (Pietri et al., 2016). Interestingly, *P*. *falciparum* soluble products induce the expression of ILPs in *An. stephensi*, through both the MEK/ERK and PI3K/Akt branches of IIS and inhibiting *P. falciparum* development *in vivo* by affecting mosquito immune effector genes (Pietri et al., 2015). This ILP-mediated inhibition of parasite development is somehow contradictory with the previous findings that human insulin could favor the parasite growth, raising the possibility that even being structurally similar, vector and host insulins can elicit distinct gut responses to *Plasmodium* infection. Moreover, Castillo et al. (2011) showed that the insulin signaling pathway can directly regulate hemocyte proliferation in *Ae. aegypti*. The same study also highlights an interesting observation regarding blood-feeding: resistance and tolerance to the same bacterial pathogen dramatically change due to blood meal digestion and/or mobilization of resources for reproduction (Castillo et al., 2011).

The PKC pathway is part of this complex signaling network that responds to infection in different vectors. Inhibition of PKC blocks West Nile virus entry in C6/36 cells by inhibiting endosomal sorting (Chu et al., 2006). In *Ae. aegypti*, activation of PKC by the heme released during the digestion of blood decreases ROS production and allows an increased proliferation of indigenous microbiota (Oliveira et al., 2011a). However, the effect of PKC pathway on viral infection has not been investigated until now. PKCs have also been shown to be expressed in the midgut epithelia of *An. gambiae* and *An. stephensi* after a blood meal. As in *Ae. aegypti*, the *An. stephensi* PKC activation was also linked to the decrease of midgut epithelial barrier, resulting in the greater development of *P. falciparum* oocysts (Pakpour et al., 2013b), without modulation of NF-κB-dependent immune factors, thus indicating that the regeneration of the midgut epithelium is essential for infection control, as also suggested by Taracena et al. (2018).

So far, in spite of the advances discussed here, it remains widely unclear how this network of nutritional signaling pathways reflects on the parasite/host interaction. This reinforces the need to build a more holistic view of the interplay between metabolic changes and the canonical immune responses to vector transmitted pathogens that occur after a blood meal, using the integrative conceptual framework of immunometabolism.

## Peritrophic Matrix

The mammalian gastrointestinal epithelium is protected by the secretion of a mucus layer, mostly composed of highly glycosylated proteins (mucins) (Johansson et al., 2013; Sicard et al., 2017). The hydrophilic O-linked oligosaccharides that coat these proteins give them the physical and chemical properties that support the mucus protective role. Mucins are secreted by specialized cells, such as goblet cells in the intestine, and have a short half-life, which ensures the constant renewal of the mucus barrier (Deplancke and Gaskins, 2001). This barrier is essential to the digestive tract as it protects the tissue from mechanical damage by food particles, chemical aggression (pH and action of digestive enzymes) and limits direct contact with the microbiota. Although frequently neglected or ignored, insect gut also presents a bonafide mucous layer, with transcriptomic data revealing abundant expression of mucins in the midgut (Terra et al., 2018). The peritrophic matrix (PM) is an extracellular structure found in the intestinal lumen of insects that is ascribed a major role as a protective layer in the midgut, usually described as analogous to the vertebrate mucus. The PM was first described by Lyonet in 1762 and is composed of glycosylated proteins embedded in chitin fibers (for reviews see Lehane, 1997; Terra, 2001, Terra et al., 2018). PMs can be classified into two different types: Type 1 is secreted by all epithelial cells as a continuous gel-like structure that completely packages the food bolus and is formed in response to feeding. The type 2 PM is a membranous structure characterized by its constitutive secretion by a midgut region called cardia and delimits an area between the epithelium and the PM, the ectoperitrophic space. It has a tubular morphology and lines the whole gut epithelium (Shao et al., 2001). Adult mosquitoes such as *Ae. aegypti* and *Anopheles* sp. secrete type 1 PM after a blood meal but present the type 2 PM at larval stages. The same is observed in the sandfly *L. longipalpis* (Secundino et al., 2005). *Glossina* spp. and *Drosophila* present the type 2 PM also in the adult stage. Hemipteran insects like the Chagas’ disease vectors are an exception in that they lack the classical PM and the lipidic perimicrovillar membranes are responsible for the PM functions (Terra, 2001; Hegedus et al., 2019). In the case of ticks, the PM is a chitin-containing extracellular layer that covers the digestive cells, displaying a very distinct morphology to that of a typical insect PM (Matsuo et al., 2003; Grigoryeva, 2010; Kotsyfakis et al., 2015), probably reflecting their peculiar tick digestive physiology, based on intracellular digestion of host blood proteins (Lara et al., 2005).

In most insects, the PM functions as a molecular sieve, controlling the traffic of molecules between the intestinal epithelium to the lumen, compartmentalizing the digestion and protecting the epithelium from potentially cytotoxic molecules (Billingsley and Rudin, 1992). However, several of these roles have been scarcely investigated in blood-feeding insects such as mosquitoes. For example, it has been shown that disruption of PM by the action of exogenous chitinases increases the blood digestion rate, an unexpected effect that was attributed to augmented access of intestinal proteases to the blood bolus (Villalon et al., 2003). Also, the *Ae. aegypti* PM binds heme released during the digestion of hemoglobin, which was hypothesized to reduce its oxidative potential (Pascoa et al., 2002).

In contrast, the PM acting as a barrier that limits exposure to the microbial world has been more thoroughly investigated. In mammals, the relative lack of an exuberant immune response against the intestinal microbiota has been attributed to the mucus acting as a barrier that avoids direct contact between the microbiota and the epithelium and not to the microbiota subverting the host immune response, a phenomenon named as “immunological ignorance” by Hooper (2009). Thus, immune homeostasis is attained largely by the mucus layer limiting the exposure of enterocytes to the microbiota (Li et al., 2018). Similarly, in insects, the secretion of the PM approaches this pivotal immune barrier function, as it compartmentalizes the microbiota and its immune elicitors, avoiding the overexposure of the gut epithelium (Buchon et al., 2009; Kuraishi et al., 2011, 2013; Weiss et al., 2014). In this way, it was demonstrated that *An. gambiae* mosquitoes express a heme peroxidase, which is essential to crosslink the PM proteins/mucins, supporting the correct assembly of this barrier. Once this peroxidase is knocked down, the PM barrier is compromised and the gut epithelia is exposed to microbial elicitors that over activate the intestinal immune system (Kumar et al., 2010).

When the microbiota and the PM layer barrier function become unbalanced, the intestinal cell homeostasis is rapidly affected. Both in mosquito and *Drosophila* the gut stem cell populations respond to biotic and abiotic injury and their activation is prevented by the PM presence (Micchelli and Perrimon, 2006; Buchon et al., 2013; Janeh et al., 2017; Taracena et al., 2018). In *Ae. aegypti*, when the PM structure is compromised by inhibition of chitin synthesis, the epithelial midgut is exposed to the microbiota, which in turn activates the generation of ROS, causing tissue damage and leading to a regenerative response based on mitotic proliferation of progenitor cells (Taracena et al., 2018). Complementary to this mechanism, it was shown in *Anopheles* mosquitoes that genes related to the synthesis of chitin and peritrophins – that together form the structural backbone of the PM – have their expression stimulated by proliferation of the intestinal microbiota (Rodgers et al., 2017). This effect closely recapitulates the mammalian response of goblet cells that prompt the secretion of stored mucus after exposition to native and pathogenic bacteria (Coconnier et al., 1998; Deplancke and Gaskins, 2001).

In mosquitoes, the intestinal microbiota experiences an explosive expansion after a blood meal. Of course, this microbial blooming is fueled by the sudden increase in the availability of nutrients, jumping from a few thousand bacterial cells in sugar-fed *Ae. aegypti* to a plateau of a few million cells in a single midgut by 12 h after blood-feeding (Oliveira et al., 2011a). However, this is probably well below the microbial population that could be supported by an unrestrained growth of bacteria in about 2 ml of blood, posing a question that has not yet been properly addressed: how fine-tuning regulation of the microbial growth is attained? At least one of such mechanisms is the production of ROS by the gut epithelium, as already mentioned above (Ha et al., 2005a; Oliveira et al., 2011a). Consistently, it was shown that gut ROS production is also attenuated by the barrier function of the PM (Taracena et al., 2018), highlighting the existence of mechanisms that balance the microbe-killing mucosal response in a way that is neither too detrimental to the host nor to the microbiota. Of interest, this mode of operation can also be relevant during infection by pathogenic microorganisms.

The first encounter between the insect host and a vectored parasite coincides with the blood digestion in the midgut, exactly when the PM is formed, making it plausible that this immune barrier can regulate the infection’s success. Anopheline mosquitoes ingest *Plasmodium* gametocytes that will fertilize and generate the ookinetes, which will invade the intestinal epithelium by secreting chitinases activated by mosquito digestive enzymes, allowing the parasite to traverse the PM. The invasion will happen around 24 h after feeding, concomitantly with PM formation peak (Huber et al., 1991; Shahabuddin et al., 1993). Moreover, it has been shown that proteins present in the matrix can function as anchors for the parasite, promoting its penetration process (Zhang et al., 2015). Although one would expect the PM might impose a barrier to the parasite infection in the gut, several reports showed instead that the absence of this structure decreases the gut parasitemia (Billingsley and Rudin, 1992; Shahabuddin et al., 1995; Baia-da-Silva et al., 2018). The PM regulation is also essential for Leishmanial infection of sandflies (Coutinho-Abreu et al., 2010). *Leishmania* parasites do not invade the epithelium but hide in the ectoperitrophic space (between the epithelium and the PM) anchored to epithelial cells, which likely protect them from the action of digestive proteases (Pimenta et al., 1997; Ramalho-Ortigao, 2010). For the *Glossina* – *T. brucei* pair, for a long time it was not understood how the parasites crossed the flies’ PM. However, recent evidence was provided that the expression of genes related to the PM formation was influenced by infection. In these studies, the authors show that *T. brucei* targets the cardia, causing the discontinuation of PM type I secretion and allowing them to invade the ectoperitrophic space (Aksoy, 2019; Rose et al., 2020).

In the Chagas disease parasite replicative stage, the *T. cruzi* epimastigotes, adhesion to the kissing bug perimicrovillar membranes seems important for their division (Gonzalez et al., 1999). Treatment of the intestinal tissue with antiserum against the perimicrovillar membrane reduces the trypanosomatid development in the vector (Gonzalez et al., 2006). As mentioned above, kissing bugs don’t have a PM but perimicrovillar membranes, which are phospholipid membranes secreted by the gut and that, analogous to the PM, define a perimicrovillar space (Terra, 1988). The *T. cruzi* epimastigotes are attached to these perimicrovillar membranes through their flagella and membrane glycoinositolphospholipids. Hydrophobic proteins located in their surface and sugar residues present in perimicrovillar membrane glycoproteins appear to be necessary for this interaction (Zingales et al., 1982; Golgher et al., 1993; Pereira-Chioccola et al., 2000; Alves et al., 2007; Nogueira et al., 2007). In *R. prolixus*, it has been shown that another function of the perimicrovillar membranes is the promotion of the aggregation of heme molecules, forming nucleation sites that convert heme into hemozoin crystals and hence preventing heme toxicity toward both the host and the parasite, that consequently creates a favorable environment for pathogen growth (Oliveira et al., 2000; Stiebler et al., 2014; Ferreira et al., 2018).

The role of the *Ae. aegypti* matrix in controlling viral infections has not been investigated so far. It is not clear whether the viral particle can get through the pores of the matrix or if the invasion of the epithelium occurs in the first hours after the meal, when the matrix has not yet been completely modeled. The latter hypothesis is the most accepted by the community, although there is a knowledge gap behind this topic (Franz et al., 2015).

In non-hematophagous insects, it is suggested that the peritrophic matrix secretion integrates the hormonal signaling, mediated by ecdysone, to the pathways downstream to nutritional sensors (Merzendorfer and Zimoch, 2003). Feeding mosquitoes with an artificial diet of low nutritional value that promotes distension of the epithelium stimulates the synthesis of a fragile and short-lived matrix, different from the robust structure observed upon blood-feeding (Dinglasan et al., 2009; Whiten et al., 2018). Thus, it is worth assessing whether the matrix synthesis is controlled by nutritional/metabolic sensors (such as the target of rapamycin, TOR and AMP-activated protein kinase, AMPK) regulated upon blood arrival, in addition to the molecules released during digestion, such as heme, hormonal signaling induced by digestion (e.g., ecdysone and ILP) or microbial community expansion.

The understanding of the PM as a barrier that coordinates the insect intestinal immune activation beyond its digestive aspect, leads to new perceptions and insights. For example, maintenance of cellular homeostasis in addition to tissue damage repair may be central to disease tolerance (Oliveira et al., 2020), while current state of literature focuses on infection resistance. Future studies are needed that address how the microbe-associated patterns are presented to the gut epithelia, and consequently how the classical immune system will be tuned and shape the parasite life history.

## Intestinal Redox Homeostasis

Historically, research on reactive oxygen species (ROS) was pushed to a central position in biology after the discovery of superoxide dismutase (McCord and Fridovich, 1969). The scene was dominated for about 30 years by the study of the role of ROS in pathologic conditions (Lambeth, 2007; Liu et al., 2018), where oxidative stress was defined as an imbalance between antioxidant mechanisms and production of ROS by several sources, including microbe-killing NADPH oxidases of immune cells. However, the discovery of the signaling role of nitric oxide (NO) in the regulation of diverse aspects of cell physiology, followed by several reports on hydrogen peroxide acting as a second messenger of several hormones and growth factors, led to a change in paradigm, with the introduction of redox signaling and redox homeostasis as novel steering concepts (Reth, 2002; Jones, 2006; Veal et al., 2007; Jones and Sies, 2015).

As already mentioned above, the digestive apparatus of most animals is also home to an abundant and specific microbial community that is now recognized as having a major and pleiotropic impact on the physiology of the metazoan host (Douglas, 2019). However, the mechanisms that control the growth rate of the intestinal microbiota are still not fully understood. Seminal studies in *Drosophila* revealed that an intestinal Dual oxidase (DUOX) produced H_2_O_2_ in response to the presence of microorganisms in the gut lumen (Ha et al., 2005a,b). Importantly, they also showed that the gut epithelium was protected from H_2_O_2_ by its dismutation to H_2_O by a heme-peroxidase that at the time was mistakenly called “Immune regulated catalase.” A DUOX enzyme found in *Ae. aegypti* mosquitoes has its activities decreased upon ingestion of a blood meal, leading to a marked reduction of ROS levels in the midgut (Oliveira et al., 2011a). Inhibition of the mosquito DUOX caused an increase in the size of the indigenous intestinal microbiota (Oliveira et al., 2011a), revealing a role of ROS metabolism in fine-tuning the symbiotic relationship between the commensal microbial community and the mosquito, in addition to its canonical immune action in the defense against pathogens.

Several other reports addressed the relation between ROS and pathogens in insect vectors. In an elegant work, Liu et al. (2016) showed that RNAi silencing of mosquito DUOX increased dengue virus replication in the midgut. Viral NS1 protein present in the host plasma enhanced susceptibility of the mosquito to DENV infection by reducing expression of DUOX, as well as of NoxM/Nox4-art (a NOX-4 homolog specific of the arthropod lineage, Gandara et al., 2017). Moreover, the iron concentration in the host’s blood was inversely correlated to the prevalence and viral load of mosquito infection by dengue virus (Zhu et al., 2019). The catalase knockdown in *Ae. aegypti* changed the mosquito’s susceptibility to DENV but had no impact on the Zika virus (ZKV) establishment. On the other hand, the ZKV viral load in the mosquito midgut was decreased by the redox imbalance promoted by down-regulation of NRF2 antioxidant transcription factor, suggesting that other components of the redox homeostasis downstream of NRF2 are involved in the control of ZKV infection (Bottino-Rojas et al., 2018). Among those, there are canonical NRF2 targets, such glutathione S-transferase and cytochrome P450, suggested to be active in maintaining tissue homeostasis during blood digestion in the mosquito (Bottino-Rojas et al., 2018, 2019). The reduction of viral load by ROS has been explained in most cases by assuming it is inflicting direct damage to the viral particle. However, experimental proof for this hypothesis is still lacking. Interestingly, an alternative mechanism emerged from the demonstration that redox imbalance and its associated cellular damage in *Ae. aegypti* and *Ae. albopictus* midgut led to increased programmed cell death, triggering a homeostatic response based on tissue-repairing mitotic activity of intestinal stem cells (Janeh et al., 2017; Taracena et al., 2018). The same report showed that this increased cellular turnover in the gut epithelia negatively impacted vector susceptibility to arbovirus infection (Taracena et al., 2018). Notwithstanding, in mosquito strains naturally refractory to viral infection, resistance was dependent on the proper recruitment of stem cells via Delta/Notch signaling pathway (Taracena et al., 2018).

In a way similar to what happens with the mosquito down-regulation of ROS production after a blood meal (Oliveira et al., 2011a), the kissing bug *R. prolixus* also decreases the intestinal ROS generation after blood-feeding (Gandara et al., 2016). However, this is not triggered by the dietary heme, as it was shown for *Ae. aegypti* (Oliveira et al., 2011a), but by the nutritional intake, which increases amino acid levels and activates the TOR pathway, impacting negatively the production of mitochondrial ROS by an yet uncharacterized mechanism (Gandara et al., 2016). Nogueira et al. (2015) showed that while high levels of H_2_O_2_ reduced growth of the *T. cruzi* epimastigote stages, low levels increased proliferation. In contrast, antioxidant molecules reduced the proliferation of epimastigotes but increased conversion to the infective trypomastigote form, revealing that ROS levels in the bug digestive apparatus have a complex regulatory role on the life cycle of the parasite.

The phlebotomine *L. longipalpis* presents an interesting illustration of how redox homeostasis in the normal gut physiology is relevant for the parasite. In the sandfly, there is a decrease in the intestinal ROS generation upon feeding (Diaz-Albiter et al., 2012), similar to *Ae. aegypti* (Oliveira et al., 2011a) and *R. prolixus* (Gandara et al., 2016). ROS levels are increased by infection with a *Serratia marcescens* strain pathogenic for the sandfly, and uric acid (an antioxidant) administration to the sugar meal increased virulence of this bacteria, revealing that the ROS-producing pathways in the gut can be controlled by immune signaling (Diaz-Albiter et al., 2012). In contrast, *Leishmania mexicana* infection does not increase ROS levels after a blood meal (Diaz-Albiter et al., 2012), but silencing the sandfly catalase or maintaining them fed in sugar meal supplemented with H_2_O_2_ decreased the intestinal parasite load. This result revealed that not only is the parasite capable of evading immune activation of ROS, but it is additionally benefited by the down-regulation of ROS levels that is part of the normal physiology of the host. However, this goes beyond simple immune evasion by the parasite, as another report, also from Dillon’s group, showed that *Leishmania* infection indeed protected flies from death by *Serratia* co-infection, but without reducing *Serratia* levels (Sant’Anna et al., 2014), strongly suggesting that the *Leishmania* is acting by triggering tolerance to disease mechanisms, thereby reducing damage and preventing fitness loss, without killing of the pathogen (in this case, the *Serratia* bacterium).

A *Plasmodium* refractory *An. gambiae* strain was appointed to have intrinsic higher levels of H_2_O_2_, which increases even more after an infectious blood meal as part of its antiparasitic defense mediated mainly by hemocytes (Kumar et al., 2003; Molina-Cruz et al., 2008) and this could be attributed to higher mitochondrial ROS production in the refractory mosquitoes (Oliveira et al., 2011b; Gonçalves et al., 2012). The antioxidant defenses induced by blood-feeding in this model seem to be in part under the control of a redox sensor, called OXR1, that regulates both the expression of antioxidant enzymes and the success of parasite infection (Jaramillo-Gutierrez et al., 2010).

*Anopheles* mosquitoes have a unique redox metabolism upon feeding and infection, described as part of the so-called “time bomb model” where the midgut invasion by the parasites triggers an epithelial response based on protein nitration, activation of peroxidases, and, consequently, apoptosis of those invaded cells (Han et al., 2001; Kumar et al., 2004). This complex intestinal response to the ookinete invasion was molecularly dissected, revealing the role of NOX5, a NADPH oxidase member, as the source of ROS. It works with a heme peroxidase to mediate the parasite nitration (Oliveira et al., 2012). While these studies point to a conventional “immune” function for NOX5, recently, the NOX5 enzymes were shown to regulate muscular function, both in mammalian blood vessel smooth muscle contraction and in the intestinal peristalsis in *R. prolixus* (Montezano et al., 2018), adding some more complexity to this scenario and bringing about the possibility of the existence of a crosstalk between physiological mechanisms and the microbiota.

The role of reactive nitrogen species (RNS) in the metabolism of hematophagous vectors was initially revealed by the demonstration that increases in NO levels limited the development of *P. falciparum* in *An. stephensi* (Luckhart et al., 1998). Subsequent works performed by the same group led to the molecular characterization of nitric oxide synthase (NOS) (Luckhart and Rosenberg, 1999) and the modulation of NOS expression during infection by factors such as the parasite’s hemozoin pigment (Akman-Anderson et al., 2007) and glycosylphosphatidylinositols (Lim et al., 2005). It was also shown that the *An. gambiae* NOS gene is controlled by Jak/Stat pathway upon infection with *P. berghei* (Gupta et al., 2009). Nitrogen reactive species have a complex chemistry, acting not only through the canonical effect of NO on cGMP formation, but also via its reaction with superoxide forming peroxynitrite. This highly reactive intermediate modifies amino acid side chains and generates derivatives such as nitrotyrosine or nitrosothiols, which are formed in the mosquito gut and are modulated by infection, having profound effects on cell signaling (Kumar et al., 2004; Gupta et al., 2005; Peterson and Luckhart, 2006; Peterson et al., 2007; Oliveira et al., 2012). While protein nitration was shown to be relevant in triggering an anti-plasmodium response (Oliveira et al., 2012), global and mechanistic analyses of nitrosative signaling on insect physiology are still scarce. More recently, it has been reported that the kissing bug *R. prolixus* produces NO in response to *T. rangeli* (Whitten et al., 2007), and NOS inhibition allowed the proliferation of *T. cruzi* parasites in the insect gut (Batista et al., 2020). Nonetheless, the role of RNS-involved pathways and their oxidative implications to parasites/virus infection of mosquitoes or other vectors is still largely unknown and exposes an important avenue for future investigation.

Another way to positively modulate intestinal ROS in mosquitoes is through native microbiota elicitors (Oliveira et al., 2011a; Xiao et al., 2017), and this can have consequences for pathogens. An *Enterobacter* strain isolated from the midgut of wild-caught mosquito was shown to decrease *P. falciparum* infection by inducing ROS generation in the gut. The infection load was restored in the presence of vitamin C (Cirimotich et al., 2011). Accordingly, in *An. gambiae*, blood digestion increases catalase expression and activity in the midgut epithelium and catalase knockdown turns the mosquito more resistant to *P. falciparum* infection (Molina-Cruz et al., 2008). In contrast, in *An. aquasalis*, infection with *Plasmodium vivax* is increased upon catalase silencing (Bahia et al., 2013). Along with the positive effect of ROS on *T. cruzi* development in triatomine bugs discussed above (Nogueira et al., 2015), this report on *P. vivax* and *An. aquasalis* highlight the complexity of the links between redox homeostasis and parasite/host relationship, which is not explained by a simplistic microbe-killing role of ROS. Even in the several reports mentioned above where ROS levels are inversely correlated with pathogen infection (such as in the *Aedes*/arbovirus), it is not completely clear how much these ROS are produced under the control of canonical immune signaling pathways and how much is derived from the “regular” physiology, such as handling of heme and iron intake, control of microbiota, muscular activity or reticulum stress.

## Reproductive Physiology and Hormonal Regulation

Vertebrate blood-feeding is a decisive evolutionary trait needed to obtain nutrients for egg development. Different species vary dramatically in their reproductive output. Some insects, like mosquitoes, are able to lay hundreds of eggs each time they take a blood meal and this feature impacts deeply their density in endemic areas (Shaw and Catteruccia, 2019). The reproductive fitness of vectors represents a promising target to prevent disease transmission because it interferes directly with the burden caused by large populations. Nonetheless, there is evidence that these organisms balance their energy investment into different life processes, often leading to fitness trade-offs between survival, immunity, and reproduction (Schwenke et al., 2016). Therefore, biological pathways essential for reproductive fitness directly or indirectly influence elements that govern vectorial capacity.

In general, it is considered that activation of immune responses decreases reproductive output in a diverse array of insects. Amongst blood-feeding vectors, parasite-induced fecundity reduction is a strategy that is evident in many vector/parasite associations (Hurd, 2003). In malaria-mosquito systems, a challenge with bacterial components or *Plasmodium* infection promotes apoptosis of follicle cells and reduces the accumulation of protein in the ovaries, as well as the number of eggs laid (Hogg and Hurd, 1995; Ahmed et al., 2002; Ahmed and Hurd, 2006; Pigeault and Villa, 2018). An immune-mediated arrest of oogenesis was also reported in other disease vectors such as the triatomine bug *R. prolixus* and tsetse flies (Hu et al., 2008; de Medeiros et al., 2009), suggesting resource allocation toward immunity to achieve recovery from infection. Besides, it is also true that reproductively active insects have reduced resistance to infection (Schwenke et al., 2016). In mosquitoes, it was shown that the same molecular processes involved in delivering blood-acquired nutrients to maturing eggs also favor the development of *Plasmodium* oocysts in the midgut and diminish the efficiency of parasite killing by the mosquito immune system (Rono et al., 2010). Recently, it was shown that transgenic *An. gambiae* mosquitoes with reduced reproductive capacity have a significantly higher malaria transmission potential, due to an increase in parasite growth rates (Shaw et al., 2020). Due to the direct implications in currently proposed control strategies (e.g., eggless mosquitoes for population suppression) and the vacancy of descriptions of resource reallocation mechanisms in other insect vector species, this subject deserves a greater deal of attention in the field.

Hormonal control is a critical mechanism for the physiological trade-off between reproduction and immunity. JH and 20-Hydroxyecdysone (20E) are key regulators of metamorphosis and reproduction in all holometabolous insects (as reviewed by Roy et al., 2018). Specifically, the balance between JH and 20E is essential for egg maturation. In most insects, increased JH levels promote egg production and provisioning and, in contrast, high 20E titers result in the resorption of immature vitellogenic eggs (Gruntenko and Rauschenbach, 2008). However, in female mosquitoes, digestion and ovarian development are physiologically integrated through a cascade of ecdysteroid signaling initiated after a blood meal (Hansen et al., 2014). Beyond the induction of synthesis and secretion of yolk protein precursors in the fat body, 20E is shown to regulate a number of additional genes that could impact parasite development in different species. In *D. melanogaster*, ecdysone triggers a precise signaling pathway shown to modulate expression levels of antimicrobial peptides and interfere with resistance mechanisms in the context of bacterial infections (Flatt et al., 2008; Rus et al., 2013). The chemical inhibition of ecdysone signaling in the blood-feeding triatomine *R. prolixus* is able to suppress cellular and humoral immune responses, disrupting gut microbial homeostasis (de Azambuja et al., 1991; Vieira et al., 2021).

Anopheline mosquitoes are a unique model for ecdysone studies due to their strict anautogeny and male transfer of 20E to females during a monandrous copulation. The mating-induced increase in oogenesis is mediated by vitellogenic lipid transporters that also facilitate *Plasmodium* development by reducing the parasite-killing ability (Rono et al., 2010). Additionally, mating affects longevity and induces changes in the midgut that can increase susceptibility to the parasite (Dao et al., 2010; Dahalan et al., 2019). In contrast, the topical application of a 20E agonist shortens lifespan, prevents mating and egg production, and significantly blocks *P. falciparum* development (Childs et al., 2016). Therefore, 20E exerts a long-range regulation of multiple physiological processes that are highly relevant to the mosquito’s competence to transmit malaria: reproductive success, parasite development, and longevity. Recently, a direct influence of 20E on cellular immune function and antipathogen immunity in mosquitoes was demonstrated. Blood-feeding of *An. gambiae* females or direct 20E injections increase phagocytic activity and this ecdysone-mediated immune priming reduces bacteria and *Plasmodium berghei* survival (Reynolds et al., 2020). However, some argue that in natural settings, the coevolution of parasite and vector has led to a less conflicting relationship, in which the immune response is toned down, and the potential cost of infection for invertebrate hosts is minimized (Mitchell and Catteruccia, 2017). Werling et al. (2019) provided evidence of a positive correlation between mosquito and parasite fitness dependent on 20E signaling. By genetic ablation of ovary development and impairment of 20E endogenous production, it was determined that ecdysone signaling is required for *P. falciparum* development via the production of mosquito host-derived lipids (Werling et al., 2019). This supports a model where the provision of lipid molecules during vitellogenesis is used by the parasite to increase survival and optimize its transmission (Costa et al., 2018). Therefore, the intricate interplay mediated by 20E between insect reproductive physiology and parasite development remains partially unresolved. It is possible that ecdysone signaling has tissue and/or threshold-specific actions, enabling the establishment of infection while boosting anti-parasite responses through distinct mechanisms. To warrant a proper impact in pathogen transmission of future discoveries, further research should ideally consider field/natural conditions and be focused on parasite-vector based combinations that occur in the wild.

Juvenile hormones control almost every aspect of insect’s life. The seminal studies of Wigglesworth, started in the early 1930s, established the existence and major roles of JH in insects, regulating tissue morphogenesis, vitellogenesis and immune response, acting primarily as an ‘inhibitory hormone’ (Wigglesworth, 1965). Additionally, strong evidence across a range of insect taxa endorse the model in which mating increases JH titers and suppresses 20E, promoting egg development and inhibiting immune capability (Schwenke et al., 2016). Much of what we know of the molecular regulation of JH in blood-feeding species comes from studies in *Ae. aegypti*. Here, the rate of JH synthesis in young stages of mosquitoes is in close correlation with their nutritional status (Noriega, 2004). JH also has essential functions in adult females where it controls post-eclosion maturation, leads to reproductive competency and ability to feed on blood, and regulates gene expression after blood-feeding (Clifton and Noriega, 2011; Roy et al., 2015). Aside from morphological alterations in the ovary, transcriptional JH-induced changes in the fat body render this tissue competent to respond to ecdysone produced by the ovary after blood-feeding (Zou et al., 2013). Moreover, JH is delivered by *Ae. aegypti* males during mating, which increases egg development by directing nutritional resources toward reproduction (Klowden and Chambers, 1991; Clifton et al., 2014). In *Drosophila*, a similar mating-induced expression of JH results in a remodeling of the female midgut, leading to cell division, increased organ size and ultimately a higher food intake (Reiff et al., 2015). However, in mosquitoes, a midgut-remodeling process in mated females has not been addressed yet. This event is relevant in disease vectors because it could be aimed to favor nutrient absorption toward egg provisioning, but also benefit pathogen development by deviating resources from immunity and supporting a higher parasite count due to an expanded gut area. JH is transported to target tissues by the hemolymph carrier juvenile hormone-binding protein (JHBP) where it binds to the methoprene-tolerant (Met) receptor and exerts its pleiotropic effects. One of the overall processes affected by Met depletion in mosquitoes is lipid metabolism (Wang et al., 2017), which could have a deep impact on the mounting of immune responses to pathogens and parasite maturation (Cheon et al., 2006). Recently, Kim et al. (2020) described a specific role for JH, through JHBP mutation, in regulating innate immune responses and the development of hemocytes in *Ae. aegypti*. JHBP-deficient mosquitoes are immunosuppressed at the humoral and cellular levels, and present a severe susceptibility to bacterial infection (Kim et al., 2020). These results call for more detailed studies exploring the role of JH signaling on vector infection by disease pathogens.

Blood-feeding initiates a complex series of physiological events in the gut, fat body and ovary that are integrated by the actions of JH, 20E and peptide hormones. Among the topics that could earn further exploration, the modulating role of sex hormones and their effects on non-reproductive organs are poorly understood in blood-feeding insects. Recently, in *Drosophila*, it was proven that an ecdysone-dependent signaling from the ovaries to the gut promotes growth of the intestine (Ahmed et al., 2020). In addition, the insect midgut produces certain hormones when it recognizes harmful components or pathogenic bacteria in an ingested meal; concurrently, these hormones regulate other tissues and organs (as reviewed in Wu et al., 2020). Given that blood digestion, parasite development and vitellogenesis require the coordination of molecular events in three different abdominal tissues, these inter-organ relationships have relevance in the context of vector–parasite interactions and deserve further attention. Furthermore, between insects of the different taxa, the diversity in life history traits may lead to distinctive adaptations of these systems (Schwenke et al., 2016) and the study of reproductive processes with respect to species-specific features can help the identification of novel targets for vector control.

## Multiple Blood-Feedings

The competence to support pathogen development varies between vectors due to many biological aspects, which include not only immunity but also feeding behavior and nutritional status (Lefèvre et al., 2013). Frequency of feeding is undisputedly an important factor in relation to human infections with insect-transmitted diseases (Kramer and Ebel, 2003; Das et al., 2017). Multiple blood meals can increase vectorial capacity by promoting the contact of the disease-carrying insect with susceptible hosts. In the study of vector biology, it is widely accepted for blood-feeding dipterans that host seeking is halted by a full blood meal (Klowden, 1990). However, a number of observations where gravid females still display host-seeking behavior motivated a reconsideration of this assumption (Scott et al., 1993; Guzman et al., 1994; Beier, 1996; Matthews et al., 2016). As most vectors of diseases need to take additional blood meals after becoming infected to complete the transmission cycle, pathogens may have evolved mechanisms to promote their success, redefining the role of uninfected blood-feedings in the epidemiology of these diseases.

One of the classic entomological parameters used in malaria transmission models rely on the proportion of bites experienced per person and the number of total bites taken by a mosquito per gonotrophic cycle (Tedrow et al., 2019). Due to their low reserves, anophelines frequently seek more than one blood meal at each oviposition cycle (Beier, 1996). This behavioral aspect of *Anopheles* females appears to increase not only fecundity, but also longevity and resistance to insecticides (Oliver and Brooke, 2017). Interestingly, the development of the malaria parasite seems to equally affect and be affected by frequent blood meals, which accelerate oocyst maturation and sporozoite development (Beier et al., 1989; Ponnudurai et al., 1989), and are induced by pathogen–vector manipulation to further enhance transmission (Cator et al., 2012). Shaw et al. (2020) proved that previously infected *An. gambiae* females, when provided a second uninfected blood meal, present an increase in oocyst growth rates and faster accumulation of sporozoites in the salivary glands, which can indeed amplify local malaria transmission potential (Shaw et al., 2020). Therefore, this previously overlooked multiple feeding behavior is a justifiable current trend of investigation in vector-borne pathogen transmission.

In the phlebotomine sand fly species *Lutzomyia*, a higher proportion of insects heavily infected can be found after the second blood meal (Moraes et al., 2018). Also, the subsequential feeding induces a faster proliferation of *Leishmania* parasite infective forms and its rapid migration to the vector proboscis, increasing vectorial competence during the second gonotrophic cycle (Elnaiem et al., 1994; Vivenes et al., 2001). Furthermore, it has been recently proven that the taking of a second uninfected blood meal by *Leishmania*-infected sand flies triggers a specialized developmental stage of the parasites, the retroleptomonad promastigotes, which is a replicative form and amplifies both the host infection and the infectiveness of the bite (Serafim et al., 2018).

As previously mentioned, blood-feeding triggers intense physiological changes to the gut tissue – including mechanical distention of the midgut, altered cell homeostasis, and changed permeability of the basal lamina – that could aid pathogen dissemination out of the midgut (Okuda et al., 2007; Dong et al., 2017; Taracena et al., 2018). Indeed, in the mosquito *Ae. aegypti*, stretching of the gut tissue over consecutive blood-feedings seems to be a critical factor causing the midgut basal lamina to become permissive for viral escape (Kantor et al., 2018; Cui et al., 2019). Due to blood-feeding-induced microperforations in the basal lamina, virus-infected individuals fed an additional non-infectious blood meal disseminate and transmit viruses more efficiently than single-fed mosquitoes (Armstrong et al., 2020). It is interesting to note that the previous discussed transfer of male reproductive gland substances during mating in *Ae. aegypti* can increase blood-feeding frequency, potentially affecting pathogen transmission by female mosquitoes (Villarreal et al., 2018). One could simply postulate that the enhanced or accelerated parasite/pathogen development upon a sequential feeding is due to the higher availability of nutrients. However, other hypotheses can explain this phenomenon, like the aforementioned diversion of energy from the immune response to support oogenesis, and further evolutionary adaptations exploited by the parasite that favor their own development and transmission.

One of the most intriguing propositions in this field is that vector-borne parasites directly manipulate phenotypic traits of their vectors and hosts in ways that increase the contact between them, hence favoring transmission. Major observational examples include *Plasmodium*, *Leishmania*, and *Trypanosoma* spp. manipulating the behavior of mosquitoes, sand flies and kissing bugs, respectively (Hurd, 2003). Frequently studied changes include alterations of biting rates in vectors and increased attractiveness of vertebrate hosts (Lefèvre and Thomas, 2008). Moreover, interesting direct evidence suggests that parasite infection reduces the insect feeding efficiency, prolonging probing time, thus enhancing the likelihood of infecting multiple hosts during a single feeding cycle (Stierhof et al., 1999; van den Abbeele et al., 2010). According to this hypothesis, in malaria-mosquito systems, vector manipulation by the parasites decreases vertebrate host seeking during the pre-infectious phase, lowering the risk of mosquito death during early parasite development. Once the vectors have become infectious, these proceedings are again increased (Schwartz and Koella, 2001). Hence, mosquitoes harboring transmissible sporozoites would be more likely to bite several people per night (Koella et al., 1998). However, most of the evidence of manipulation comes from avian or rodent model systems and is focused on isolated components of mosquito host-feeding process (e.g., host detection, probing, piercing, blood ingestion and terminating the feed) (Friend and Smith, 1977). Therefore, the complexity of human malaria models makes it difficult to characterize how infection affects this multiple set of behaviors (Cator et al., 2012). Furthermore, when long evolutionary association between specific *Plasmodium* and *Anopheles* species combinations are tested, distinct or null alterations are shown (Nguyen et al., 2017; Stanczyk et al., 2019). Overall, behavioral manipulations stand as a complex phenomenon that continues to require careful observations with the use of different methods and multidisciplinary approaches (Lefèvre et al., 2006).

Regardless of the mechanism, vector-borne disease transmission depends on the frequency at which the insect vector bites humans. Therefore, fundamental investigations on the impact of complex vector behaviors, and of the ecology and evolution of vector–pathogen interactions, remain key aspects needed to generate better predictions of disease transmission and of the efficacy of control interventions. The varied transmission strategies evolved by pathogens and the vectors’ behavioral changes induced by them can affect how current control tools work. Moreover, biting frequency and how bites are distributed among different people also can have significant epidemiologic effects (Woolhouse et al., 1997; Cooper et al., 2019). High multiple-feeding rates can explain why reducing vector populations alone is difficult for prevention and support the argument for additional studies on feeding behavior (Harrington et al., 2014). Given that multiple blood-feedings directly increase the number of potentially infective encounters, this impact should be considered on model predictions and, accordingly, shape specific vector control strategies. This could mitigate the possibility of underestimating transmission intensity, which could lead to a misunderstanding of the impact of vector control (Tedrow et al., 2019).

## Concluding Remarks

The growing impact of vector-borne diseases urgently calls for the development of new entomological interventions. Increasing knowledge on insect biology and insect–pathogen interactions that help unravel the processes that determine vectorial capacity will fuel innovative approaches to stop transmission (Shaw and Catteruccia, 2019). Insect hosts can resist infection or limit/tolerate the deleterious effects caused by the pathogen. Tolerance refers to all host defense mechanisms that limit ‘damage to functions and structures’ during infection, without interfering with pathogen load, as defined more than 60 years ago by plant pathologists (Caldwell et al., 1958). Therefore, resistance has a negative effect on pathogen fitness, whereas tolerance does not. The genetic trade-off between resistance and tolerance can shape the successful evolutionary interactions in a vector-pathogen system (Lambrechts and Saleh, 2019; Oliveira et al., 2020).

**FIGURE 1:**
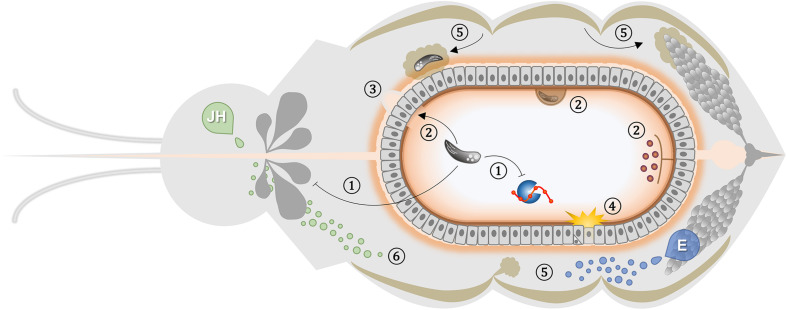
Generic representation of non-immune elements that affect vector-pathogen interaction. The scheme depicts a frontal plane of a blood-feeding insect showing the relative position and interactions of the different non-immune players involved in diverse parasite-vector associations. Organ and cell sizes are not up to scale. **(1)** Parasites can manipulate feeding rate and digestion activity of the host (Stierhof et al., 1999; Sant’Anna et al., 2009). **(2)** Peritrophic matrix regulates intestinal infection, requiring parasite escape to establish invasion (Huber et al., 1991; Rose et al., 2020); allowing parasite anchoring to ectoperitrophic space (Pimenta et al., 1997; Zhang et al., 2015); and compartmentalizing microbiota to ensure immune ignorance of the epithelium (Weiss et al., 2014). **(3)** Blood-feeding-induced microperforations in the basal lamina support pathogen dissemination (Kantor et al., 2018; Armstrong et al., 2020). **(4)** Rupture of the peritrophic matrix barrier activates ROS generation that triggers an epithelial response to infection (Oliveira et al., 2011a; Taracena et al., 2018). Juvenile Hormone (JH) and Ecdysone (E) are key regulators of the physiological trade-off between reproduction and immunity. **(5)** Ovary ecdysone production exerts paramount effects such as fat body-induced provision of lipid molecules during vitellogenesis that can reduce the parasite-killing ability and support its development (Rono et al., 2010; Werling et al., 2019). **(6)** Juvenile hormone influence diverse physiological processes in the insect that can impact pathogen success such as ability to feed on blood, midgut remodeling, reproductive competency, gene expression regulation, lipid metabolism and immune response mounting (Clifton and Noriega, 2011; Zou et al., 2013; Roy et al., 2015; Kim et al., 2020).

Here, we strengthen the argument that the insect vector response to infection does not merely activate immune pathways as a mechanism of resistance. It also encompasses a broad range of adaptive consequences, including metabolic alterations, stress responses, and tissue repair. Many of these are related to blood-feeding and reproduction (Figure 1). These events can lead to improved survival of the insect despite active pathogen replication. The impact of infection on the vector can thus be tuned by the parasite to favor both physiological host homeostasis and completion of the transmission cycle. Moreover, the target for natural selection is seldom one isolated organ or a discrete event, but rather are the multisystemic processes that involve pathogen acquisition and development within the vector. Despite recent advances in the knowledge of physiological mechanisms that can work as non-canonical immunological traits, several elements have yet to be unfolded. Future novel vector control strategies may arise rooted in integrated system biology research to target physiological aspects that act as protective mechanisms and contribute to tolerance to infection. In this way, innovative and effective tools can be used, in an evolutionary considerate manner, to mitigate the great burden imposed on societies by vector-borne diseases.

## Author Contributions

All authors listed have made a substantial, direct and intellectual contribution to the work, and approved it for publication.

## Conflict of Interest

The authors declare that the research was conducted in the absence of any commercial or financial relationships that could be construed as a potential conflict of interest.

## Abbreviations

TG: triacylglycerol
TGF β: transforming growth factor-beta
ILP: insulin-like peptides
PI3K: phosphoinositide 3-kinase
PKC: protein kinase C
ERK: extracellular signal-regulated kinase
MEK: MAPK/ERK kinase
NF κ B: nuclear factor kappa-light-chain-enhancer of activated B cells
NRF-2: nuclear factor erythroid 2–related factor 2
OXR1: oxidation resistance protein 1
cGMP: cyclic guanosine monophosphate.
